# Nature's nitrite-to-ammonia expressway, with no stop at dinitrogen

**DOI:** 10.1007/s00775-021-01921-4

**Published:** 2021-12-05

**Authors:** Peter M. H. Kroneck

**Affiliations:** Department of Biology, University of Konstanz, Universitätsstrasse 10, 78457 Konstanz, Germany

**Keywords:** Ammonium, Cytochrome *c*, Cytochrome *c* nitrite reductase, Multiheme enzyme, Nitrite, Nitrogen cycle

## Abstract

**Abstract:**

Since the characterization of cytochrome *c*_552_ as a multiheme nitrite reductase, research on this enzyme has gained major interest. Today, it is known as pentaheme cytochrome *c* nitrite reductase (NrfA). Part of the NH_4_^+^ produced from NO_2_^−^ is released as NH_3_ leading to nitrogen loss, similar to denitrification which generates NO, N_2_O, and N_2_. NH_4_^+^ can also be used for assimilatory purposes, thus NrfA contributes to nitrogen retention. It catalyses the six-electron reduction of NO_2_^−^ to NH_4_^+^, hosting four His/His ligated *c*-type hemes for electron transfer and one structurally differentiated active site heme. Catalysis occurs at the distal side of a Fe(III) heme *c* proximally coordinated by lysine of a unique CXXCK motif (*Sulfurospirillum deleyianum*, *Wolinella succinogenes*) or, presumably, by the canonical histidine in *Campylobacter jejeuni*. Replacement of Lys by His in NrfA of *W. succinogenes* led to a significant loss of enzyme activity. NrfA forms homodimers as shown by high resolution X-ray crystallography, and there exist at least two distinct electron transfer systems to the enzyme. In γ-proteobacteria (*Escherichia coli*) NrfA is linked to the menaquinol pool in the cytoplasmic membrane through a pentaheme electron carrier (NrfB), in δ- and ε-proteobacteria (*S. deleyianum*, *W. succinogenes*), the NrfA dimer interacts with a tetraheme cytochrome *c* (NrfH). Both form a membrane-associated respiratory complex on the extracellular side of the cytoplasmic membrane to optimize electron transfer efficiency. This minireview traces important steps in understanding the nature of pentaheme cytochrome *c* nitrite reductases, and discusses their structural and functional features.

**Graphical abstract:**

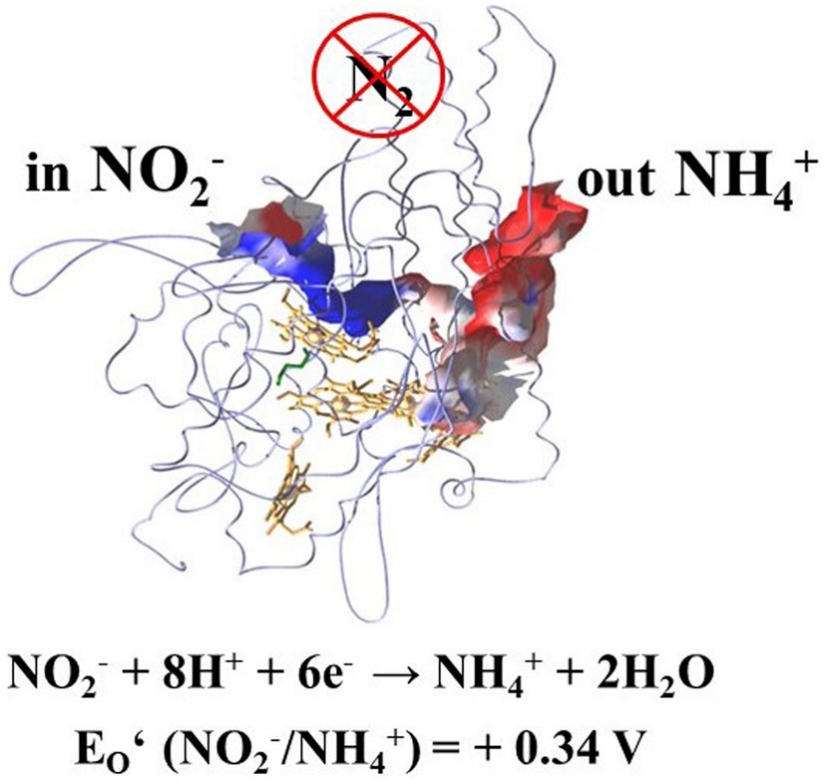

## Introduction

The focus of this minireview is on multiheme *c*-type cytochromes, written in honor of Isabel Moura and former SBIC president José Moura (2010–2012) from the Universidade Nova de Lisboa in Portugal on the occasion of their 70th birthday. In my view this topic seems well suited, considering the numerous important discoveries and contributions by Isabel and José to our understanding of transition metal enzymes, many of them key components of the global nitrogen cycle, carrying Fe, Cu, or Mo ions at the active site [1–16]; for a complete list of their publications please visit https://sites.fct.unl.pt/biologicalchemistryatfctunl/pages/people accessed on 30 Nov 2021.

**Fig. 1:**
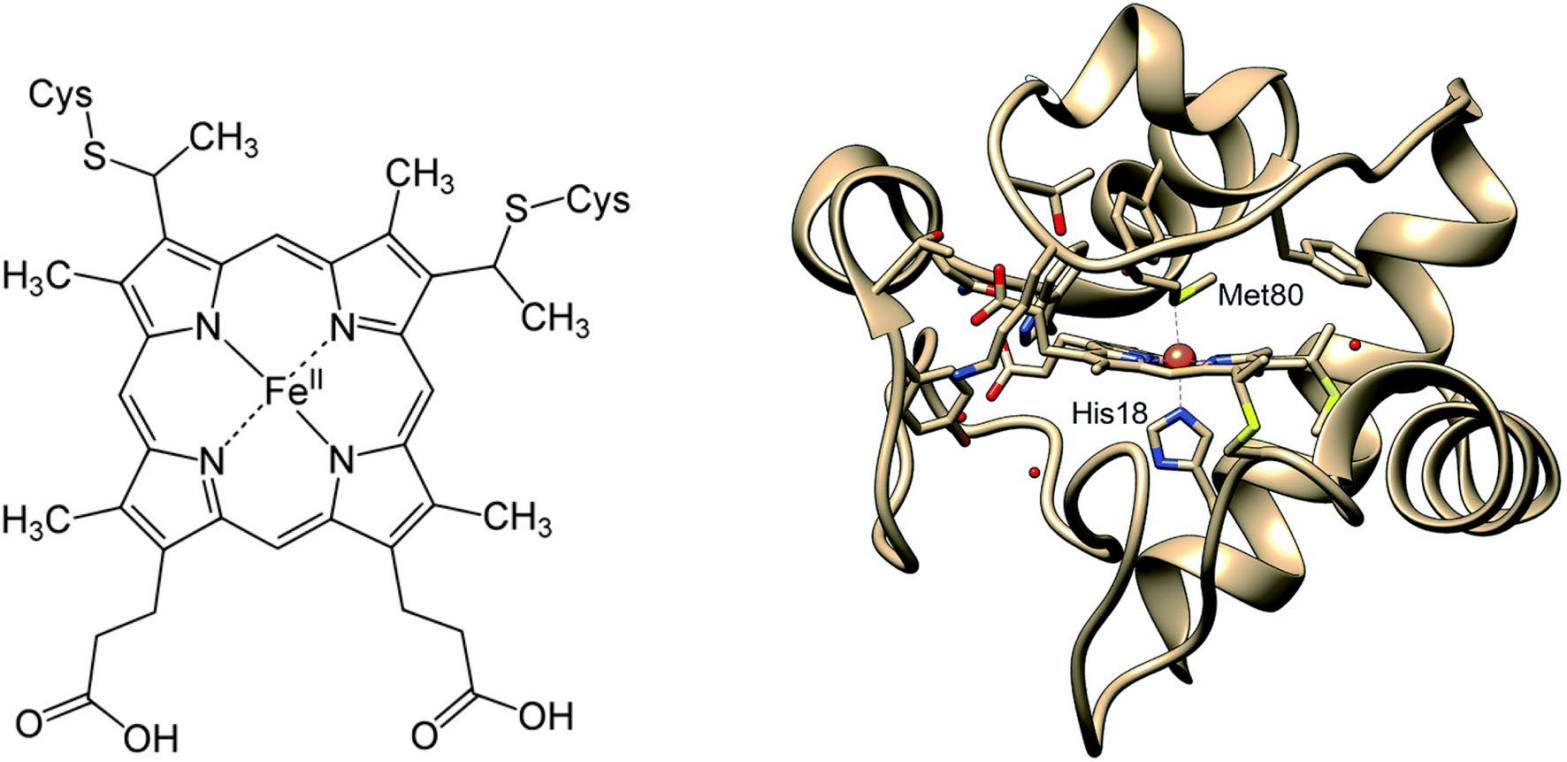
Left: structure of heme *c*, with the covalently attached heme group present in cytochromes *c*. Right: structure of cytochrome *c* (PDB 1HRC), with Met80 and His18 ligated to iron [20]

In *c*-type cytochromes the heme is covalently attached to the polypeptide backbone via two thioether (R–S–R′) bonds formed by the vinyl groups of heme and cysteine side chains in a Cys-X-X-Cys-His pentapeptide motif; “X” denotes a miscellaneous amino acid, and the histidine residue coordinates on the proximal binding site of the heme iron (Fig. 1). Cytochromes *c* possess a wide range of properties, they function as electron transfer proteins and are involved in many important redox processes [17–35].

In this contribution I will center on the pentaheme enzyme cytochrome *c* nitrite reductase (NrfA), a key player within the global nitrogen cycle [36–46]. It catalyses the six-electron reduction of nitrite (NO_2_^−^) to ammonium (NH_4_^+^) (Eq. 1), as part of the dissimilatory nitrate reduction to ammonium (DNRA) process, that competes with denitrification [47–57]. Notably, NrfA can also convert sulfite (SO_3_^2−^) to hydrogen sulfide (H_2_S), an important reaction of the microbial sulfur cycle (Eq. 2), performed by dissimilatory sulfite reductase employing the coupled siroheme-[4Fe-4S] center for catalysis [58–62].

1 $$^{{ + {\text{III}}}} {\text{NO}}_{{2}}^{ - } + {\text{6e}}^{ - } + {\text{8H}}^{ + } {\to}^{{ - {\text{III}}}} {\text{NH}}_{{4}}^{ + } + {\text{2H}}_{{2}} {\text{O}}\;\left( {E_{0}^{\prime } + 0.{34}0{\text{ V}}} \right), $$

2 $$^{{ + {\text{IV}}}} {\text{SO}}_{{3}}^{{{2} - }} + {\text{6e}}^{ - } + {\text{8H}}^{ + } \to {\text{H}}_{{2}}^{{ - {\text{II}}}} {\text{S}} + {\text{3H}}_{{2}} {\text{O}}\left( {E_{0}^{\prime } {-}0.{\text{117 V}}} \right). $$

**Scheme 1:**
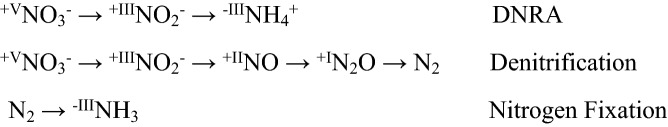
Key processes of the global nitrogen cycle: dissimilatory nitrate reduction to ammonium (DNRA), denitrification, and nitrogen fixation; changes in the oxidation state of nitrogen, as well as in equations, are indicated by roman numerals

Unlike the process of denitrification, DNRA, also known as nitrate/nitrite ammonification, conserves bioavailable nitrogen in the system, producing soluble NH_4_^+^ rather than chemically unreactive dinitrogen gas (N_2_) (Scheme 1) [47–57].

With the report by Fujita on soluble cytochromes in *Enterobacteriaceae* in 1966, followed by the purification and description of cytochrome *c*_552_ as hexaheme nitrite reductase in 1986 [63–65], the stage was set for an all-out attack on the problem of microbial nitrite to ammonia reduction by numerous pioneering researchers. Among them Isabel and José Moura, who applied advanced spectroscopic (EPR, Mössbauer) and electrochemical techniques to unravel structural and functional properties of these complex multi-centered metalloenzymes [1, 6, 8, 10], see also recent review entitled “How Biology Handles Nitrite” [54]. Here I will give an account of my personal experience in this exciting field of Bioinorganic Chemistry, and I will mention some of the great moments in this endeavour. In view of the vast literature in the field of *c*-type cytochromes, I suggest for introduction the monographs by Ambler [18], Pettigrew and Moore [19], and Salgueiro and Dantas [31], for deeper insight into the complex topic, the informative articles by experts are recommended [21–35]. My apologies go to all colleagues who made significant contributions to our current knowledge of cytochrome *c* nitrite reductases and related enzymes that will not be discussed here. These omissions are not intentional, they are the consequence of time and space. Clearly, the emphasis is on the structural and functional properties of the pentaheme nitrite reductases from *Sulfurospirillum deleyianum* and *Wolinella succinogenes*. Yet this minireview will hopefully illustrate what one may learn about studying the structure and function of such an important and intensively studied enzyme.

## Looking back: Tomar 1979—Jean Le Gall—cytochrome ***c***_3_—***Desulfovibrio***

I did my Ph.D. work under the supervision of Peter Hemmerich [66] at the newly founded University of Konstanz, in an area of research nowadays called Bioinorganic Chemistry [67–74]. Hemmerich had fruitful collaborations with scientists from around the world: Helmut Beinert (Madison), Anders Ehrenberg (Stockholm), Jean-Marc Lhoste (Paris), Vincent Massey (Ann Arbor), Israel Pecht (Rehovot), Jack Spence (Logan), and Cees Veeger (Wageningen), to name a few. Advanced spectroscopic techniques, among them magnetic resonance methods as well as stopped-flow and rapid quench kinetics in the millisecond range, were established in Konstanz and applied to investigate the structure and function of complex flavin and metal-dependent enzymes. I started my work with two plant proteins, the blue multi-copper enzyme ascorbate oxidase and the type 1 Cu protein mavicyanin together with Augusto Marchesini (Milan) [75–77].

**Fig. 2:**
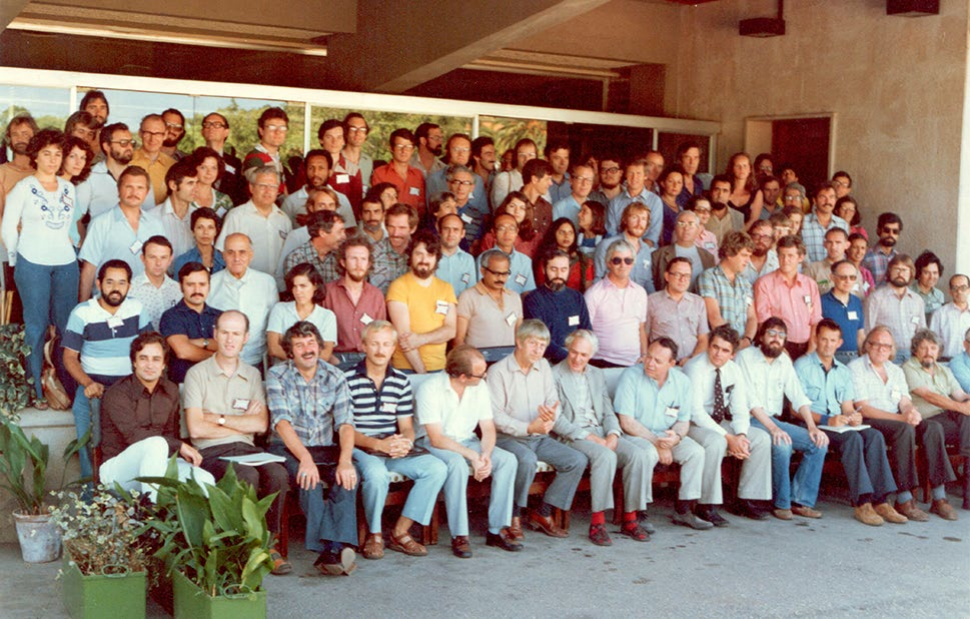
Participants of the workshop “Metal Ions in Biology”, sponsored by NATO Advanced Study Institutes, held September 16–28, 1979 in Tomar, Portugal [79]. The photograph shows António Xavier, Allen Hill, Isabel and José Moura, Helena Santos, Isabel Coutinho, Jean Le Gall, and numerous pioneers of Bioinorganic Chemistry mentioned in the text

In 1979 I attended a workshop in Tomar (Portugal) entitled “Metal Ions in Biology”, sponsored by NATO Advanced Study Institutes, and organized by António Xavier (Lisbon) and Allen Hill (Oxford) (Fig. 2). It was in Tomar where I met Isabel and José Moura for the first time. Both, together with Helena Santos and Isabel Coutinho, were working hard to keep us participants happy. Clearly, this meeting became a memorable milestone in the history of Bioinorganic Chemistry. Four years later Ivano Bertini (Florence), Harry Gray (Pasadena), Bo Malmström (Göteborg), and Helmut Sigel (Basel) initiated the International Conference on Biological Inorganic Chemistry (ICBIC) series in Florence [78, 79].

For 2 weeks, leading researchers presented their work on metal-dependent enzymes in Tomar. In parallel, distinguished experts offered excellent—however quite exhausting—lectures on physical techniques, such as magnetic circular dichroism (MCD), Mössbauer, nuclear magnetic resonance (NMR), and electron paramagnetic resonance (EPR) spectroscopy, and not to forget the application of direct electrochemical methods to study electron transfer (ET) and catalysis [79]. I recall a crude three-dimensional model presented by Richard Haser and colleagues (Marseille) for the structure of the tetraheme ET protein cytochrome *c*_3_. The protein had been isolated from *Desulfovibrio desulfuricans*, and its structure was solved at 2.5 Å resolution [80–82]. The molecule consisted of a single polypeptide chain wrapped around a compact core of four non-parallel heme centers. Alignment of the amino acid sequences of cytochrome *c*_3_ from different sources suggested that the structure reported by Haser and colleagues was characteristic of the cytochrome *c*_3_ group which became the target of numerous NMR studies by Xavier and his associates [26, 83–86]. Notably, in the course of our early spectroscopic and structural studies of metal-dependent proteins and enzymes in sulfate reducing bacteria, we isolated cytochrome *c*_3_ from the periplasmic fraction of *D. desulfuricans* (strain Essex 6) and Oliver Einsle solved its three-dimensional structure. Its major physiological function appeared to be that of an electron carrier for the periplasmic hydrogenase, and Julia Steuber provided evidence that it interacted with the membrane-associated dissimilatory sulfite reductase (DSIR). In addition, a nonaheme cytochrome *c* from this organism could be structurally characterised by Günter Fritz in Konstanz [87–90]. Last but not least, the structural origin of nonplanar heme distortions in Fe(III) cytochromes *c*_3_ was investigated by resonance Raman spectroscopy together with John Shelnutt (Albuquerque), Isabel and José [91].

Next, I have to mention microbiologist Jean Le Gall from the Laboratoire de Chimie Bacterienne (Marseille) whom I met in Tomar for the first time. He also held a position as Research Professor of Biochemistry and Microbiology at the Department of Biochemistry, University of Georgia (Athens) [92, 93]. As a graduate student he discovered a new species of bacteria which he named *Desulfovibrio gigas* (*gigas*, latin, meaning *gigantic*). These bacteria are so-called anaerobes and extremely difficult to grow. Clearly, Jean Le Gall has to be regarded as one of *the* pioneering researchers in the field of Inorganic Microbial Sulfur Metabolism, and he turned into one of the most influential collaborators of Isabel and José Moura, and António Xavier and associates [94–107].

Admittedly, when I was trained as a chemist at the Universities of Basel and Konstanz in the 1960- and 70-ties (with a strong preference for transition metal coordination chemistry), anaerobic bacteria of the genus *Desulfovibrio*, microbial bioenergetics as well as biogeochemical cycles of the elements nitrogen and sulfur were not part of the program. It was not until 1986, when Andreas Zöphel finished his doctorate thesis on microbial sulfur respiration by “Spirillum 5175”. In Lisbon, supported by the Gulbenkian foundation, Andreas learned how to handle anaerobic bacteria and how to isolate and purify dioxygen sensitive enzymes. “Spirillum 5175” uses the reduction of elemental sulfur (S^0^) to hydrogen sulfide (H_2_S) for energy conservation (sulfur respiration). Together with Norbert Pfennig, Professor of Microbiology in Konstanz, and graduate student Wolfram Schumacher, “Spirillum 5175” was described as the type strain of the new genus and species *S. deleyianum* [108–110]. Thanks to Norbert Pfennig and his associates, Heribert Cypionka, Bernhard Schink, and Friedrich Widdel, and inspired by pioneering researchers of Microbiology and Bioinorganic Chemistry, either through experiments in the laboratory, or by fruitful discussions at international conferences, new and fascinating areas of research with exciting discoveries arose. Enzymes with novel transition metal centers (Fe, Cu, Mo, W) and unique catalytic properties could be purified from both anaerobic and aerobic microorganisms. These metal-dependent enzymes, among them two multi-centered key players of the global nitrogen and sulfur cycle, cytochrome *c* nitrite reductase and dissimilatory sulfite reductase, were structurally characterized and their mechanism of action was investigated by applying biochemical and spectroscopic methods [111, 112].

To finish my backward glance, close to four decades after the Tomar meeting, in 2015, both José and I were invited to participate in the workshop “Feeding the World in the twenty-first Century: Grand Challenges in the Nitrogen Cycle”. The workshop was initiated by Nicolai Lehnert (University of Michigan), with co-organizers Gloria Coruzzi (New York University), Eric Hegg (Michigan State University), Lance Seefeldt (Utah State University), and Lisa Stein (University of Alberta). In short, the purpose of this workshop was to identify ways that chemists can help the scientific community understand and manage the nitrogen cycle to improve agriculture and environmental quality [113].

## Multiheme proteins and enzymes

**Fig. 3:**
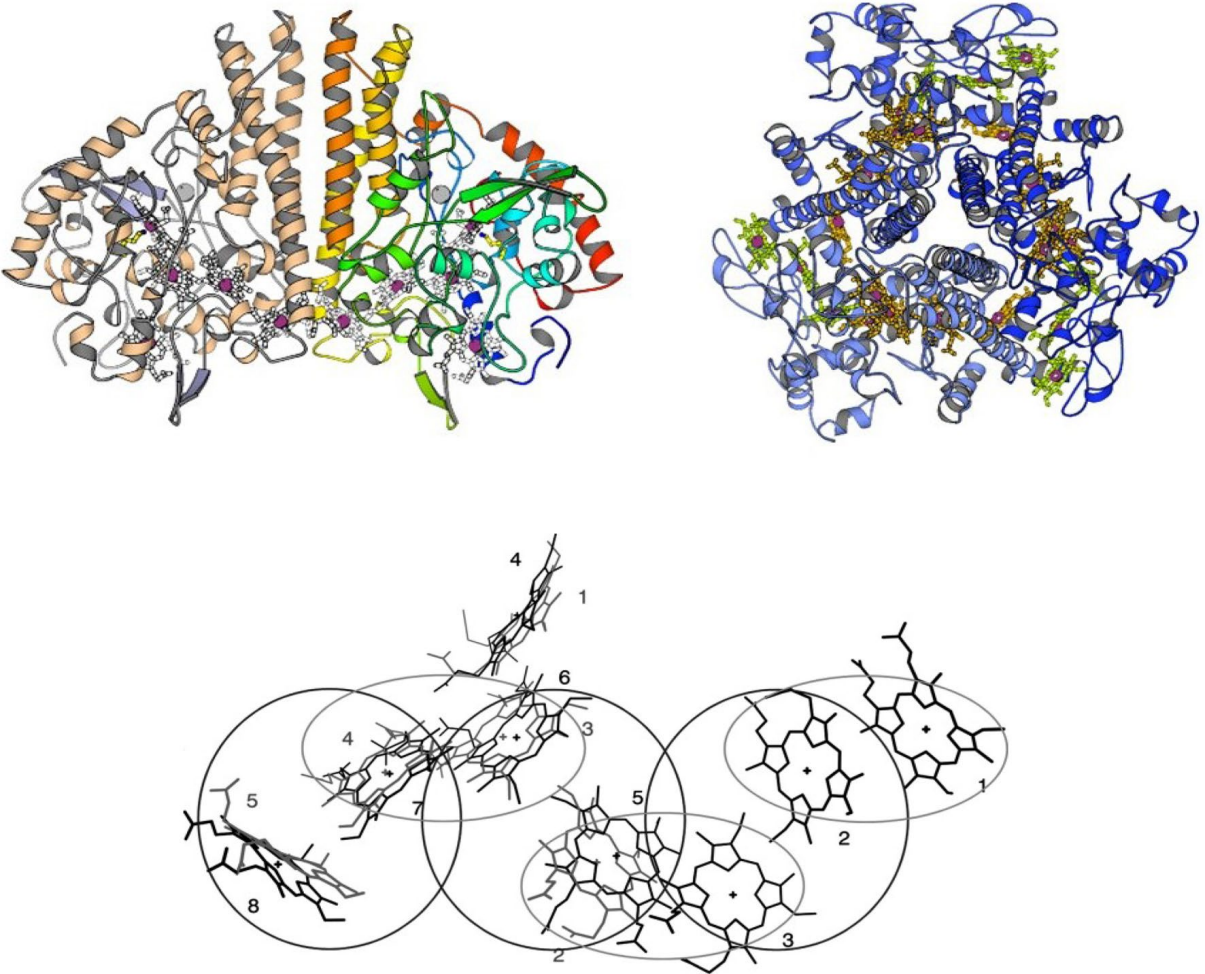
Structural comparison of pentaheme cytochrome *c* nitrite reductase of *W. succinogenes* (NrfAws) and octaheme hydroxylamine oxidoreductase of *Nitrosomonas europaea* (HAO). Left: NrfAws, functional homodimer; PDB 1FS7). Right: HAO, functional homotrimer; PDB 1FGJ). Below: Superposition of the heme groups of NrfAws (gray) and of HAO (black) numbered according to their attachment to the protein chain. With the exception of the active site heme (one in NrfA, four in HAO), all heme groups form so-called di-heme elbow motifs (circles), which are connected via a parallel stacking arrangement like in split-Soret cytochrome *c* (ovals) [104, 123, 163]

Heme proteins (Fe protoporphyrin IX complexes) exhibit an impressive range of biological functions, such as ET reactions, dioxygen (O_2_) transport and storage, O_2_ reduction to hydrogen peroxide (H_2_O_2_) or water (H_2_O), and the oxygenation of organic substrates (R–H → R–OH). The range of functions can be extended further by linking heme groups with other redox active cofactors and metal sites plus other heme centers. These combinations will permit heme cofactors to couple ET with other processes, such as the translocation of protons or the reduction/oxidation of molecules both inside and outside of the cell. In aerobic and anaerobic microbes, especially those of the biological nitrogen and sulfur cycles, there are many *c*-type cytochromes with multiple heme centers per polypeptide chain [2, 3, 5, 23, 25–32, 35, 55, 98, 103, 114–122]. Early examples include the octaheme hydroxylamine oxidoreductase (HAO) and related proteins [123–129], the tetraheme NapC/NirT/TorC family [130], the 16-heme-containing protein Hmc (high molecular mass cytochrome *c*) from sulfate-reducing bacteria [131, 132], the octaheme tetrathionate reductase from *Shewanella oneidensis* [133, 134], the tetraheme cytochrome *c*_3_ described in the previous section, and the pentaheme cytochrome *c* nitrite reductase, the central enzyme of this review. These multiheme proteins form structurally related families, in which the positions of the heme can often be overlaid, even when there is little sequence conservation between members of the family, e.g., pentaheme nitrite reductase NrfA, octaheme HAO, and flavocytochrome fumarate reductase [22, 133–135]. In 1999, Barker and Ferguson argued that only by fixing the hemes spatially via their thioether bonds can such clustering of hemes be achieved [22]. Furthermore, the dense packing of hemes allows rapid electron transfer between the heme centers [136], an essential part of the function of these cytochromes. This molecular arrangement is advantageous for enzymes such as NrfA, which catalyses the six-electron reduction of nitrite (Eq. 1), or the group of cytochrome P460 enzymes, which catalyse the oxidation of hydroxylamine (NH_2_OH) [45] (Fig. 3).

Finally, one of the recent important scientific discoveries in the global nitrogen cycle has to be brought forward, the anammox process (Anaerobic Ammonium Oxidation) described by Gijs Kuenen and associates (Delft) in 1995 [137, 138]. On a global scale, anammox bacteria significantly contribute to the removal of fixed nitrogen from the environment [139]. The process depends on multiheme proteins that structurally resemble the ones known from other microorganisms, but that exhibit new functions. In the three-step process, NH_4_^+^ becomes oxidized by anaerobic ammonium-oxidizing bacteria, they can oxidize ammonium with nitrite as the oxidant instead of O_2_ and form N_2_ as the end product (Eqs. 3–6).

3 $$^{{ + {\text{III}}}} {\text{NO}}_{{2}}^{ - } + {\text{2H}}^{ + } + {\text{e}}^{ - } {\to}^{{ + {\text{II}}}} {\text{NO}} + {\text{H}}_{{2}} {\text{O}}\;\left( {\Delta G^{{{\text{o}}\prime }} \, = - {113}.{\text{38 KJ}}/{\text{mol}}; + 0.{\text{347 V}}} \right), $$

4 $$^{{ + {\text{II}}}} {\text{NO }} {+}^{{ - {\text{III}}}} {\text{NH}}_{{4}}^{ + } + {\text{ 2H}}^{ + } + {\text{ 3e}}^{ - } {\to}^{{ - {\text{II}}}} {\text{N}}_{{2}} {\text{H}}_{{4}} + {\text{ H}}_{{2}} {\text{O}} \left( {\Delta {\text{G}}^{{{\text{o}}\prime }} \, = - {116}.{\text{27 KJ}}/{\text{mol}}; + 0.{\text{126 V}}} \right), $$

5 $$^{{ - {\text{II}}}} {\text{N}}_{{2}} {\text{H}}_{{4}} \to {\text{N}}_{{2}} + {\text{4H}}^{ + } + {\text{4e}}^{ - } \left( {\Delta {\text{G}}^{{{\text{o}}\prime }} = - {128}.{1}0{\text{ KJ}}/{\text{mol}}; - 0.{\text{746 V}}} \right), $$

6 $$^{{ + {\text{III}}}} {\text{NO}}_{{2}}^{ - } {+}^{{ - {\text{III}}}} {\text{NH}}_{{4}}^{ + } \to {\text{N}}_{{2}} + {\text{2H}}_{{2}} {\text{O}} \left( {\Delta {\text{G}}^{{{\text{o}}\prime }} = - {\text{357 KJ}}/{\text{mol}}} \right). $$

Notably, substrate conversion proceeds through potentially toxic intermediates nitric oxide (NO) and hydrazine (N_2_H_4_). The anammox machinery resides in a special and unique cell organelle, the Anammoxosome. Here, energy released in the anammox reaction is used to drive ATP synthesis, powered by novel membrane-bound protein complexes [140, 141]. The end product N_2_ is produced from the oxidation of intermediate N_2_H_4_, with the octaheme protein hydrazine dehydrogenase (HDH) involved in catalysis which appears to be related to octaheme HAO. HDH is a soluble multi-protein complex (1.7 MDa) that is not spatially associated with the anammoxosome membrane. The enzyme of *Kuenenia stuttgartiensis* was characterized as a covalently cross-linked homotrimeric octaheme protein. The HDH trimers build an octameric architecture, with each octamer harbouring an amazing 192 (!) *c*-type heme centers. It is concluded that the multi-protein complex observed both X-ray crystallography and Cryo-Electron Microscopy, probably represents the functionally relevant oligomeric state of HDH [142–146].

## Sulfur respiration and reduction of nitrite to ammonia in *Sulfurospirillum deleyianum* and *Wolinella succinogenes*

**Fig. 4:**
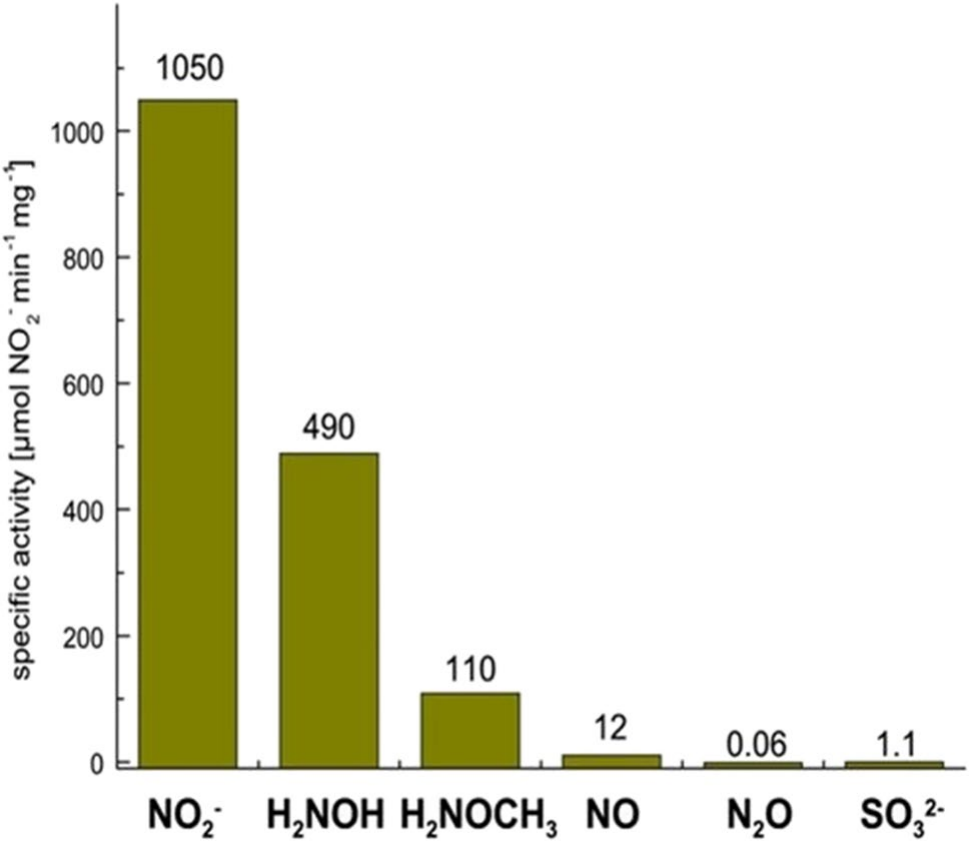
Specific nitrite reductase activity and substrate spectrum of cytochrome *c* nitrite reductase of *S. deleyianum* (NrfAsd). With nitrogen monoxide (NO), hydroxylamine (NH_2_OH), and O-methyl hydroxylamine (NH_2_OCH_3_) ammonium (NH_4_^+^) was the product; in the case of nitrous oxide (N_2_O), dinitrogen (N_2_) was formed most likely; with sulfite (SO_3_^2−^) hydrogen sulfide (H_2_S) was the only product [60, 156]

When asking myself how I, a coordination chemist by training, got into the field of metal-dependent proteins and enzymes in aerobic and anaerobic microorganisms, clearly all my excursions into microbiology are linked, from the early experiments on sulfur respiration to cytochrome *c* nitrite reductase and multiheme *c*-type cytochromes, with Norbert Pfennig. Together with Regina Bache, a survey was made of components of sulfur-reducing bacteria that can be detected by EPR spectroscopy around 10 K [147]. Among the organisms investigated was a small spirillum later described as *S. deleyianum*, which turned out to be a model organism for (1) studying sulfur respiration, that is the reduction of elemental sulfur (S^0^) to hydrogen sulfide (H_2_S), and (2) exploring the structural and functional characteristics of DNRA enzyme cytochrome *c* nitrite reductase (NrfA) [39, 43, 44, 108–112, 147–157]. Basically, DNRA is a short circuit that bypasses the processes of denitrification (NO_3_^−^ → N_2_) and nitrogen fixation (N_2_ → NH_3_) (Scheme) [155]. Dihydrogen (H_2_) and formate (HCOO^−^) are the predominant electron donors for various nitrite-ammonifying bacteria including *S. deleyianum* and *W. succinogenes*. Sulfide (S^2−^) can serve as electron donor as well, thus connecting the biogeochemical cycles of nitrogen and sulfur [150]. In this case, the presence of a highly active nitrite reducing system might help to dispose toxic NO_2_^−^. When grown with NO_3_^−^, NO_2_^−^, or S^0^ as terminal electron acceptor, *S. deleyianum* expressed a red protein with intense EPR resonances centered at *g* ≈ 3.85 and 9.12 (recorded in perpendicular mode) and at *g* ≈ 9.8 (recorded in parallel mode), localized mainly in the membrane fraction. Under reducing conditions (Na^+^ dithionite) the prominent cytochrome in the membrane fraction exhibited absorption maxima at 553, 522.5 and 426 nm, and notably, both the soluble and the membrane fraction of *S. deleyianum* showed high nitrite reductase activity [108, 109, 147]. Following the intense EPR signal at g 3.85, Wolfram Schumacher isolated a heme-dependent nitrite reductase from both the soluble and membrane fraction of *S. deleyianum* and related microorganism *W. succinogenes*; it reduced NO_2_^−^ to NH_4_^+^ with a specific activity of up to 1050 µmol NO_2_^−^ (protein min)^−1^ (Fig. 4) which was key to build a nitrite sensor based on a highly sensitive nitrite reductase mediator-coupled amperometric detection [151–153]. The UV/Vis spectrum of the enzyme was typical for *c*-type cytochromes with absorption maxima at 280, 408, 532 and 610 nm (oxidized) and at 420, 523 and 553 nm (reduced). The EPR spectrum (perpendicular mode) revealed resonances at g 9.82, 3.85, 3.31, 2.95, 2.30, and 1.49, resulting from high-spin and low-spin Fe(III) heme centers, as reported for other nitrite reductases purified from *D. desulfuricans*, *W. succinogenes*, or *Escherichia coli* [151]. Early on, the characteristic signal at g 3.85, which could also be observed in EPR spectra of whole cells of *S. deleyianum* grown with S^0^, was suggested to originate from a magnetic interaction between high-spin and low-spin Fe(III) hemes [158].

**Fig. 5:**
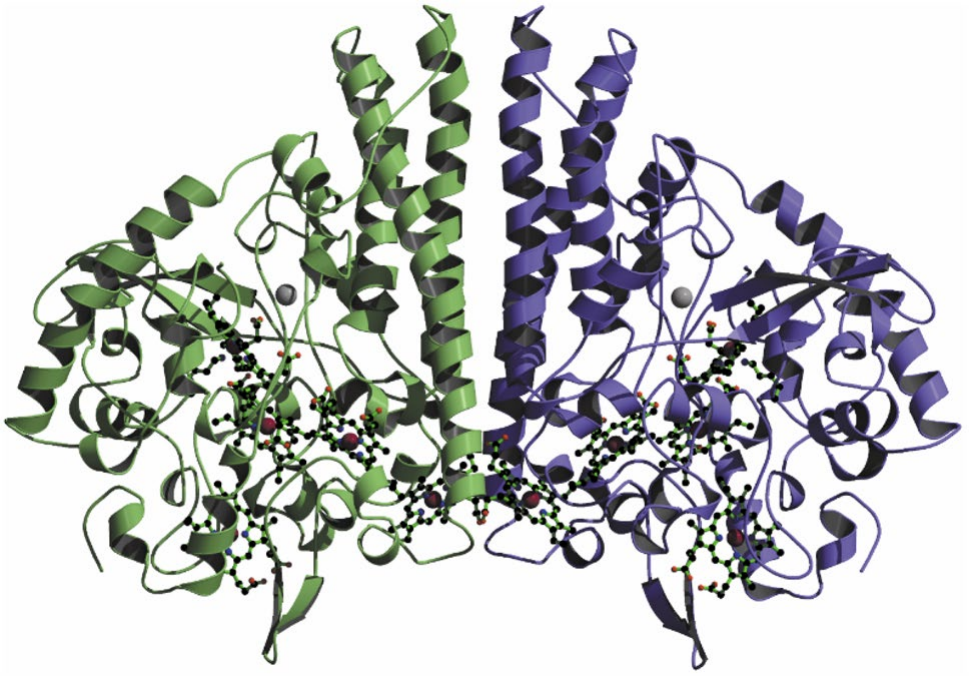
Overall structure of the cytochrome *c* nitrite reductase dimer (NrfAsd PDB 1QD8; NrfAws PDB 1FS7); front view of the dimer, dimer formation is mediated by the central helical segments. The peptide chain packs into a compact, predominantly α-helical fold that can be subdivided into the central part, where long helices form the dimer interface, and the heme containing part, where the peptide chain wraps tightly around the cofactors; Fe ions in red, heme groups in green, Ca^2+^ in grey close to active site heme; the five hemes in the monomer are in close contact, with Fe–Fe distances of between 9.3 and 12.8 Å, and 11.7 Å for hemes at dimer interface [162–164]

The view of the existence of a family of hexaheme nitrite reductases, based on their similar sizes, specific activities, UV/Vis and EPR spectral properties, had to be abandoned in 1993 when the amino acid sequence of the *E. coli* enzyme became available. During this time both Jeff Cole (University of Birmingham) and I had suggested, based on sequence and spectroscopic data, that NrfA of *E. coli* [159, 160] as well as of *S. deleyianum* and *W. succinogenes* hosted only four *c*-type hemes each [152]. Notably, the *E. coli* NrfA sequence revealed the presence of just ten cysteine residues, eight arranged as four conventional CXXCH heme-binding motifs. However, in a consecutive publication, Cole and associates provided convincing evidence for a fifth heme covalently attached to the remaining two cysteine residues within a novel cysteine-lysine Cys-Trp-Ser-Cys-Lys motif, and that the lysine residue is required for normal rates of nitrite to ammonia reduction [161]. Oliver Einsle, together with Albrecht Messerschmidt and Robert Huber (Max Planck Institute of Biochemistry, Martinsried), gave the final answer to the question about the number of hemes and nature of heme binding motifs by solving the three-dimensional structure of cytochrome *c* nitrite reductases of *S. deleyianum* (1.9 Å resolution) [162] and of *W. succinogenes* (1.6 Å) [163]. The molecular architecture showed a homodimer with five hemes anchored in each subunit, four of them bound to the protein with the conventional Cys-X-X-Cys-His motif, and one with the novel cysteine-lysine Cys-X-X-Cys-Lys motif (Fig. 5) [164]. These characteristic basic features of NrfA, detected in nitrite reductase of *S. deleyianum* and *W. succinogenes*, have been also found in the NrfA structures of *E. coli* [165, 166], *D. desulfuricans* ATCC27774 [167, 168], *S. oneidensis* [169], the NrfA_4_H_2_ complex of *D. vulgaris* [170, 171], and most recently in NrfA of the bacterium *Geobacter lovleyi* [172, 173].

However, recent investigations of the diversity and phylogeny of the NrfA enzyme, analysing 272 full-length NrfA protein sequences, distinguished 18 NrfA clades, with 3 clades having the conventional CysX-X-Cys-His motif in the first heme-binding domain, whereas all other clades had the Cys-X-X-Cys-Lys motif in this location [174, 175]. Earlier studies on the ε-proteo-bacterium *Campylobacter jejeuni* did already indicate that the putative *nrfA* gene carried the conventional Cys-X-X-Cys-His motif, and not the Cys-X-X-Cys-Lys motif present in *E. coli*, *S. deleyianum*, or *W. succinogenes* [176].

## Structure of pentaheme cytochrome *c* nitrite reductase

Note that the main focus will be on the structural and functional properties of the cytochrome *c* nitrite reductase of ε-proteobacteria *W. succinogenes* (NrfAws) and *S. deleyianum* (NrfAsd); for a more comprehensive analysis the article by Oliver Einsle in Methods of Enzymology is recommended [164].

### Overall structure and protein architecture

Both NrfAsd (PDB 1QD8) [162] and NrfAws (PDB 1FS7) [163] are pentaheme enzymes encoded by a single gene termed *nrfA*, with a protoporphyrin IX covalently linked to the protein backbone at the active site. The protein forms a homodimer with dimensions of ≈ 100 Å × 80 Å × 50 Å, in which the ten covalently attached heme groups are closely packed (Figs. 3, 5). It folds into one compact domain, with α-helices as the predominant secondary structural motif, ranging from short helical turns to four long helices at the carboxy-terminal end of the peptide chain; ß-sheet structures are only found in two short antiparallel strands, where one is part of a funnel-like cavity leading to the active site. The dimer interface is dominated by three long α-helices in each monomer, and although the area of this interface amounts to only ≈ 10% of the total surface area of the protein, the arrangement is highly conserved. The five heme groups in each monomer are sufficiently close to assure rapid intramolecular electron transfer that also bridges the dimer through the adjacent heme group 5 from each monomer. Heme center 1 is the active site of the enzyme, showing the typical scheme found in *c*-type cytochromes, with thioether bonds to the Cys residues of the heme binding motif. It has a Lys residue to replace the conventional His as a proximal ligand to the heme iron. The Fe atom is coordinated by the N_ζ_ atom (sp^3^ N) of Lys (Fe–N distance 2.1 Å/NrfAws), and the distal axial position remains open for interaction with the substrate. As expected from amino acid sequence comparisons, the structures of NrfA from both *S. deleyianum* [162] and *W. succinogenes* [163] are very similar (root mean square displacement for C_α_ atoms 1.19 Å). The main differences in the peptide chain are two insertions in NrfAsd, which are absent in the NrfAws sequence. Apart from these insertions and the additional residues at the N terminus of the NrfAws polypeptide chain, a further major difference is the C-terminus of the chain, which forms an α-helix in NrfAsd and a short two-stranded β-sheet in NrfAws. The residues that surround the active site and form the substrate/product channel are completely conserved [162–164].

In many multiheme *c* proteins, heme groups pack into distinct and recurring motifs that were recognized early on in electron transfer proteins, e.g., cytochrome *c*_554_ from *N. europaea* [177] and enzymes, such as NrfA. They were classified in particular into a parallel and a perpendicular stacking interaction (Fig. 3). These two types of interactions are combined in the proteins to form efficient electron transfer chains. The five hemes in the NrfA monomer are in close contact, with Fe–Fe distances of between 9 and 12.8 Å. They are arranged as a group of three, almost coplanar hemes (1, 3, 4), with heme 1 forming the active site. Hemes 2 and 5 are farther apart and are not coplanar with hemes 1, 3 and 4. All hemes, with the exception of heme 1, are His/His coordinated; with edge-to-edge distances below 4 Å, hemes 1, 3 and 4 are close enough to allow direct π-electron interaction of the porphyrin rings. The propionate side chains of heme 1 form part of the active-site cavity, while those of heme 4 are exposed to the solvent and those of heme 3 are hydrogen-bonded inside the protein. All porphyrins show a slight distortion from planarity, most strongly in heme 2, and least in the active-site heme 1. Heme 2 could function as the entry point for electrons, see discussion on the complex between NrfA and its redox partner. The dimer interface is dominated by three long α-helices per NrfA monomer. Hemes 5 interact across the dimer interface which is even closer than hemes 2 and 3 within each monomer. The short Fe–Fe distance of 11.7 Å will allow efficient electron transfer across the NrfA dimer interface [136] which might be functionally relevant. Furthermore, both these hemes interact directly through hydrogen bonds between their propionate side chains. Note that the relative orientation of the five heme groups in NrfAsd corresponds exactly to the one observed in NrfAws, including the fact that heme 1 is the five-coordinate active site heme group, clearly all the structurally and functionally important features are conserved between both species. The five heme groups of NrfAws align with those of NrfAsd within a root mean square displacement of 0.12 Å for all atoms [162–164].

Fe–Fe distances between the heme groups are in a range commonly found in redox proteins and are short enough to allow for direct electron tunneling between the individual heme centers [136]. Whereas heme 1 is the site of nitrite binding, it is more difficult to define the entry point for electrons delivered by the physiological redox partner, the tetraheme *c*-type cytochrome NrfH [178, 179]. All heme groups cluster on one side of the dimer, and heme 2 as well as heme 5 have one edge of the porphyrin plane exposed to the solvent, although for heme 5, most of this area is in the dimer interface and is covered upon dimerization. Furthermore, the area where heme 2 reaches the protein surface is located within a patch of strong positive surface potential in the NrfAws structure. In *S. deleyianum*, the membranous nitrite reductase complex was described to be less stable than in *W. succinogenes* [152], and in accordance with this, the positive patch surrounding heme 2 is less pronounced. Thus, heme 2 has been proposed to be the most likely entry point for electrons into the nitrite reductase of both *S. deleyianum* and *W. succinogenes* [162–164]. The electropositive patch in the vicinity of heme 2 is conserved in the *E. coli* enzyme (NrfAec), however, it is significantly less pronounced than in NrfAsd and NrfAws. The size of the positive patch is reduced in the NrfAec structure by the substitution of an adjacent arginine residue (Arg 207/NrfAws; Arg 206/NrfAsd) for a glutamine residue (Gln 205). Although weak, this represents one of the few positive surface patches apart from that at the site of substrate entry to heme 1. This weakness may relate to the observation that, it has not been possible to isolate and crystallize stable complexes of NrfAsd, NrfAws, or NrfAec with its electron donors, either NrfH or NrfB, and to solve their three-dimensional structures [152, 165, 180]. A significant step forward in understanding the complex architecture of the complete cytochrome *c* nitrite reductase machinery came from the work by Inês Pereira, Margarida Archer, and associates (Lisbon) who solved the X-ray structure of the stable complex between the reductase NrfA and its electron donor NrfH from *Desulfovibrio vulgaris*. One NrfH molecule interacts with one NrfA dimer in an asymmetrical manner, forming a large membrane-bound complex with an overall α_4_ß_2_ quaternary arrangement. The menaquinol-interacting NrfH heme is pentacoordinated, bound by a methionine from the CXXCHXM sequence, with an aspartate residue occupying the distal position. The NrfH heme that transfers electrons to NrfA has a lysine residue from the closest NrfA molecule as distal ligand [170, 171]. 

**Fig. 6:**
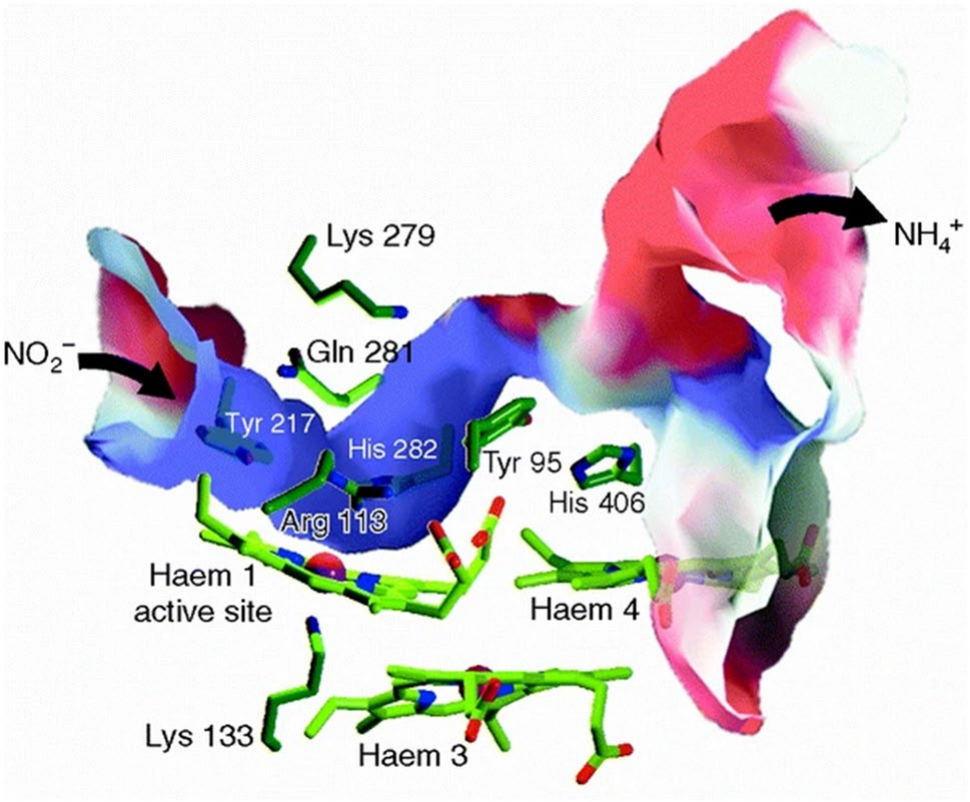
The active-site channel of cytochrome *c* nitrite reductase of *Sulfurospirillum deleyianum* (NrfAsd PDB 1QD8). Apart from the channel (entrance) guiding NO_2_^−^ from the protein surface to the catalytic heme 1 site, a second channel (exit) reaches the protein surface on the opposite side of the molecule. The whole channel is coloured according to the electrostatic surface potential, blue for a positive and red for a negative potential. In contrast to the immediate surroundings of the active-site heme group 1, the surface potential is drastically changed in the presumed exit channel for NH_4_^+^ [162]

Cytochrome *c* nitrite reductase does not only convert NO_2_^−^ to NH_4_^+^, but also the potential reaction intermediates NO and NH_2_OH, as well as N_2_O, NH_2_OCH_3_, and SO_3_^2−^ (Fig. 4). However, intermediates are not released during nitrite turnover. Obviously, the active site cavity can accommodate both anions and uncharged molecules, and will release the NH_4_^+^ cation only after the full six-electron reduction of NO_2_^−^. The preference for anions is reflected by a positive electrostatic potential around and inside the active site cavity, induced by the residues forming the cavity, Tyr 127, His 282, Arg 113, Gln 281, and Lys 279 (numbering NrfAsd). These residues serve as stores for protons required for the reduction of NO_2_^−^ to NH_4_^+^ (Eq. 1) and can be resupplied by water molecules. Considering the good accessibility of the active site for H_2_O and the presumably lower pH on the periplasmic side of the cytoplasmic membrane, the product of NO_2_^−^ reduction will be the NH_4_^+^ cation rather than uncharged NH_3_. The cationic product might take advantage of a second channel leading to the protein surface opposite to the entry channel. This second channel is lined by His 406 and Tyr 95 and filled with coordinated water molecules, it branches before reaching the protein surface and ends with both arms in areas possessing a significantly negative electrostatic surface potential (Fig. 6) [162, 163].

### Active site

**Fig. 7:**
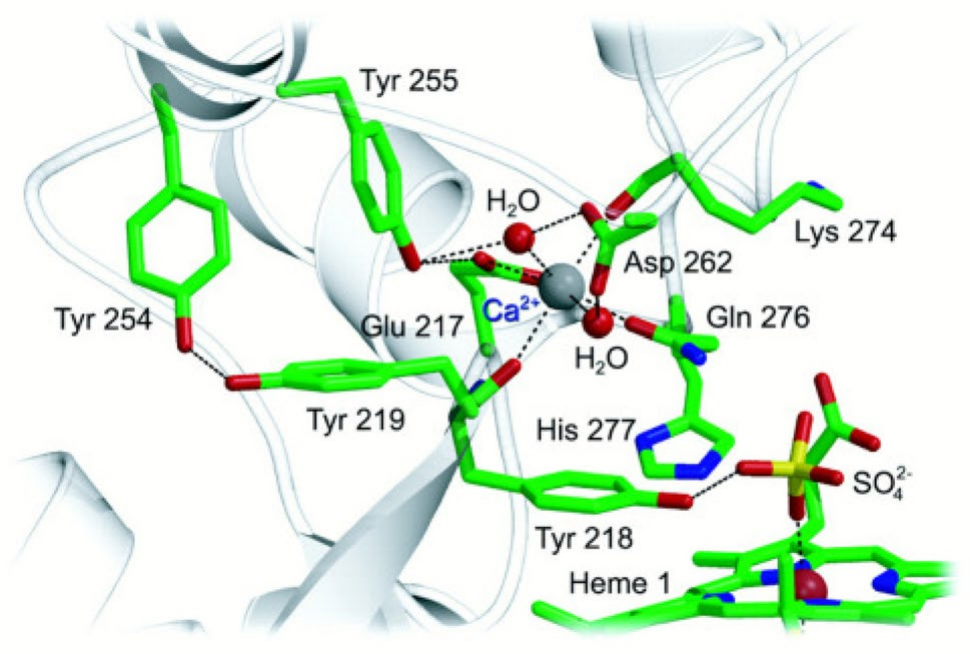
The conserved Ca^2+^ site in cytochrome *c* nitrite reductase (NrfAsd) bridges two stretches of protein that host active site residues. Note the close proximity between heme 1 (SO_4_^2−^ bound), and a set of conserved tyrosine residues which was thought a role in radical stabilization during catalysis [162, 197]

The site of nitrite reduction is heme center 1, with the N_ζ_ atom (sp^3^ N) of lysine, replacing a His (sp^2^ N) residue in the classical binding motif, and an oxygen atom of a bound SO_4_^2−^ anion detected in the first published structures NrfAsd (Lys 133; PDB 1QD8) and NrfAws (Lys 134; 1PDB 1FS8) (Fig. 7) [162, 163]. The SO_4_^2−^ anion binds to the iron with an oxygen atom, it further interacts with both a His and a Tyr via a single oxygen atom, and with a water molecule which in turn interacts with the two propionate side chains of heme 1. At low concentrations of SO_4_^2−^ it was replaced by H_2_O at heme 1. The structure of the water-bound form (1.6 Å resolution) of NrfAws showed the oxygen atom of H_2_O bound to the Fe at a distance of 2.05 Å. Without the bulky SO_4_^2−^ anion, the imidazole moiety of His 277 moved closer to the heme iron, and a hydrogen bond (length 2.88 Å) was formed between its Nε_2_ and the H_2_O molecule at the active site. The positions of both Tyr 218 and Arg 114 remained unchanged [163]. Although the bound SO_4_^2−^ originated from the crystallization buffer, its binding at the active heme center provided early information about substrate binding. As expected both SO_4_^2−^, and structurally related PO_4_^3−^_,_ acted as weak inhibitors in the activity assay [156]. Azide, N_3_^−^, was expected to bind to the active site heme iron as a competitive inhibitor [181, 182] just like SO_4_^2−^. However, the structure of the NrfAws-N_3_^−^ complex (2.0 Å resolution) revealed a water bound to Fe at 2.05 Å, and in close proximity the N_3_^−^ anion, bound to residues lining the active site entrance, with hydrogen bonds to Gln 276 (3.0 Å), Tyr 218 (2.8 Å) and two hydrogen bonds to Arg 114. With N_3_^−^ bound in this fashion, the active site cavity can no longer accommodate SO_4_^2−^ [163].

A survey of NrfA (and variants) structures, including structures of NrfAws with H_2_O (PDB 1FS7), N_3_^−^ (PDB 1FS9), NO_2_^−^ (PDB 2E80), NH_2_OH (PDB 2E81), and SO_3_^2−^ (PDB 3BNF) bound at the active heme site, is compiled in [164]. The special feature of the active site heme 1 is its fivefold coordination, with the lysine ligand provided by the novel binding motif CXXCK, in contrast to the conventional bis-His coordination in the residual four heme centers. Early on it was suggested that the proximal lysine ligand is of crucial importance in promoting the binding of anions, and that the environment of this heme provides a reduction potential low enough to reduce the substrate NO_2_^−^ to NH_4_^+^ [183]. Earlier studies on *E. coli* NrfA (cytochrome *c*_552_) showed that the lysine residue was required for normal rates of nitrite reduction, if it was altered to histidine, the enzyme was inactive [161]. Jörg Simon and co-workers (Darmstadt) [184] constructed the NrfAK134H variant from *W. succinogenes*; the specific nitrite reductase activity of the cell homogenate, the membrane fraction, as well as of the soluble fraction and the purified NrfA obtained from *W. succinogenes* strain K134H did not exceed 40% of that of the wild-type system.

An electron-density maximum close to the active site heme 1 was assigned to a Ca^2+^ ion, which could be confirmed by inductively coupled plasma atomic emission spectroscopy (1.0 ± 0.1 Ca^2+^ atoms/NrfA monomer). The Ca^2+^ion, first discovered in NrfAsd [162], is coordinated by the carboxy group of a glutamate in a bidentate manner and by glutamine, two peptide carbonyl oxygen atoms and two water molecules. The roof of the active-site cavity of NrfAsd is formed by Phe 91, Lys 279, Tyr 95, Ala 404 and Gln 281, which coordinates the Ca^2+^ by its carboxamide oxygen such that the amino group faces the active site (Fig. 7). The Ca^2+^ binding site appeared to be an essential structural feature in the overall architecture of the enzyme, and the region surrounding this site is one of the most highly conserved parts of the whole sequence. This is easily understood for Tyr 218, which is an active site residue that can directly interact with substrate. It can be rationalized that the Ca^2+^ ligands Lys 274 and Gln 276 immediately precede another active site residue, His 277, such that the Ca^2+^ ion bridges two stretches of protein that hold key residues for catalysis. Furthermore, both Lys 274 and Gln 276 take part in forming the active site cavity, whose electrostatic surface potential is presumably essential for guiding substrate influx and product efflux. Another remarkable feature close to the active site is a set of tyrosine residues, which are conserved in NrfA sequences. Tyr 219 follows directly on the active site residue Tyr 218, whose backbone carbonyl oxygen is a calcium ligand (Fig. 7).

In cytochrome *c* nitrite reductase from *D. desulfuricans* ATCC 27774, a second calcium site was discovered by Maria João Romão in collaboration with Isabel, José, and colleagues, featuring an octahedral geometry, coordinated to propionates of hemes 3 and 4, and caged by a loop absent in the NrfAsd and NrfAws structures. The highly negative electrostatic potential around hemes 3 and 4 suggests that the main role of this Ca^2+^ ion may be structural, namely to stabilize the conformation of the additional caging loop and to influence the solvent accessibility of heme 4 [168]. The NrfA active site is similar to that of peroxidases with a nearby Ca^2+^ at the heme distal side nearly in the same location as occurs in the class II and class III peroxidases. This finding suggests that the Ca^2+^ ion at the distal side of the active site in the NrfA enzymes may have a similar physiological role to that reported for the peroxidases [185]. On the other hand, Eric Hegg, Nicolai Lehnert, and associates characterized NrfA from bacterium *G. lovleyi* (NrfAgl) which had recently been recognized as one of the key drivers of DNRA in nature. The enzyme crystallized as a dimer, but dynamic light scattering characterization suggested that NrfA remained a monomer in solution even up to ≈ 300 μM. In this context one should recall that the interface of the NrfA dimer, which is dominated by three long α-helices in each monomer, only accounts to ≈ 10% of the total protein surface (Fig. 5). As a consequence, the state of NrfA in solution (monomer/dimer) is expected to vary depending on the protein source and experimental conditions [164]. Along these lines, nitrite reductase activity resided both in the soluble and the membrane fraction during protein purification, but the distribution of both pools of protein varied strongly. While in *E. coli* virtually all activity was found in the soluble fraction [165], in *S. deleyianum* and *W. succinogenes*, both the soluble and the membrane fractions showed nitrite reductase activity [151, 152]. In the case of *D. vulgaris*, however, the majority of enzyme resided in the membrane fraction, forming a stable membrane-bound complex with an overall NrfA_4_NrfH_2_ quaternary arrangement [170].

Notably, the crystal structure of NrfAgl (2.55 Å) revealed the presence of an arginine residue in the region otherwise occupied by the Ca^2+^ ion in canonical NrfA enzymes, such as NrfAsd or NrfAws. The presence of chelating agent EDTA did not affect the activity of NrfAgl, and site-directed mutagenesis of this arginine reduced enzymatic activity siginificantly. Furthermore, phylogenetic analysis revealed four separate emergences of Arg-containing NrfA enzymes. Thus, the Ca^2+^-independent, Arg-containing NrfAgl represents a new subclass of pentaheme cytochrome *c* nitrite reductase [172, 173].

## Reaction mechanism of pentaheme cytochrome *c* nitrite reductase

Biological redox reactions classically involve the transfer of electrons one or two at a time. A limited group of reactions can be classified as multi-electron reductions, in that more than two electrons—in fact, as many as six—are transferred to an enzyme-bound substrate before product release from the site of reduction [186, 187]. Multi-electron reduction reactions occupy crucial positions in metabolism, being involved in the utilization of dioxygen as terminal electron acceptor by all organisms capable of aerobic metabolism, as well as in the biological conversion of inorganic sulfur and nitrogen compounds, e.g., SO_3_^2−^ and NO_2_^−^, for assimilatory (biosynthesis) and dissimilatory (energy conservation) purposes. For each of these reactions, enzymes exist that can catalyse the entire multi-electron transfer process without release of inorganic compounds of oxidation states intermediate between substrate and product. Furthermore, such novel types of redox reactions will be most likely associated with novel types of complex enzyme prosthetic groups. The enzymatic reductions of oxyanions SO_3_^2−^ and NO_2_^−^ occur as part of two different physiological processes, (1) in plants, fungi, and many bacteria, the reductions of SO_3_^2−^ to H_2_S/S^2−^ and NO_2_^−^ to NH_3_/NH_4_^+^ are intermediate steps in the assimilation of sulfate (SO_4_^2−^) and nitrate (NO_3_^−^), respectively, for the synthesis of S and N-containing cellular constituents, and (2) in microorganisms, the reductions of SO_3_^2−^ and NO_2_^−^ are large-scale processes associated with anaerobic respiration utilizing SO_4_^2−^ and NO_3_^−^ as terminal electron acceptors [111, 186, 187].

**Fig. 8:**
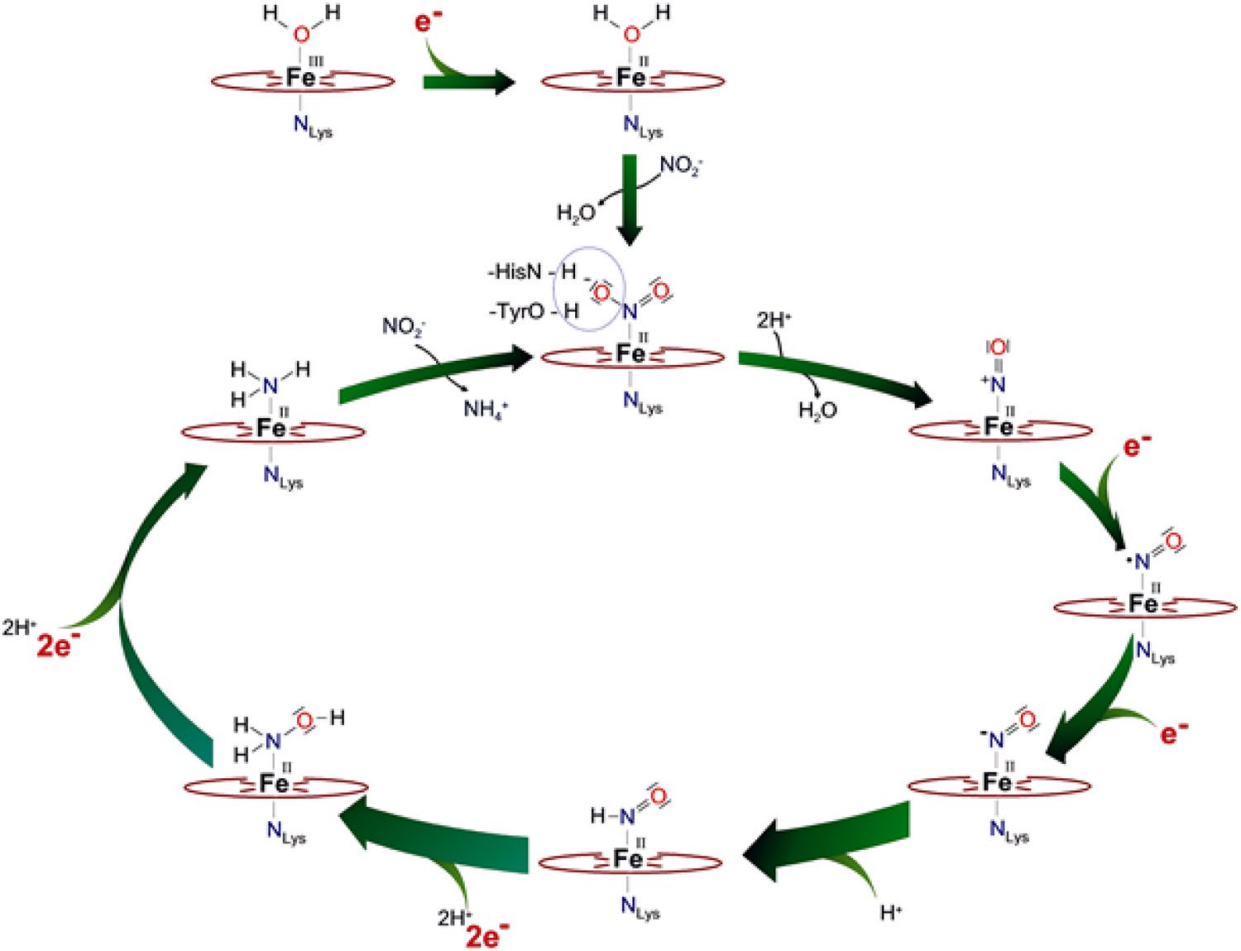
Proposed reaction scheme for the six-electron reduction of nitrite to ammonia catalysed by cytochrome *c* nitrite reductase. When started from the water-bound resting state in either the oxidized (Fe^III^) or reduced (Fe^II^) form, the binding of NO_2_^−^ starts the reaction cycle, which, after a heterolytic cleavage of the first N–O bond, proceeds through two one-electron reductions and a protonation step to Fe(II)-HNO, which is readily reduced by two electrons to Fe(II)-H_2_NOH. A further reduction leads to the dissociation of the second water molecule, and subsequently, after the last reduction step, the product ammonia can dissociate [193]. Notably, N_2_O—in contrast to NO and NH_2_OH not an intermediate in the proposed cycle—is a substrate of NrfA, however with low activity (Fig. 4), and the reaction product has not been identified so far [156]

The six-electron reductions of nitrite to ammonia/ammonium and sulfite to hydrogen sulfide/sulfide (Eqs. 1, 2) are fundamental to early and contemporary life. These multi-electron, multi-proton transfer processes are catalysed by a group of diverse nitrite and sulfite reductases that provide a unique prosthetic group assembly in their active centers with structural features that are key for the catalytic mechanism [39, 46, 188–192]. Cytochrome *c* nitrite reductase (NrfA) catalyses the reduction of NO_2_^−^ to NH_4_^+^ with high specific activity (Eq. 1), without the release of bound intermediates NO and NH_2_OH as discussed below (Fig. 8). Electrons usually are delivered from the membranous quinone pool, thereby generating a proton motive force [34].

In addition to NO_2_^−^, NrfA converts NH_2_OH and NO to NH_4_^+^ (Fig. 4) [156], furthermore, the NO reductase activity of NrfA has been later shown to play an important role within the oxidative and nitrosative stress defense network of bacteria such as *E. coli* and *W. succinogenes* [54, 55]. Note that NH_2_OH and methyl derivatives reacted poorly with Fe(III)-NrfA, on the other hand, NH_2_NH_2_ acted as reductant. Photochemically reduced NrfA (using 5-deaza-10-methyl-3-sulfopropyl-isoalloxazine/ oxalate) was rapidly oxidized by NH_2_OH and derivatives in solution, but not by NH_2_NH_2_ [157]. Attempts to produce crystalline Fe(II)-NrfAsd from oxidized Fe(III) crystals, by reaction with either Na^+^ dithionite, or dihydrogen in the presence of traces of [Ni,Fe] hydrogenase from *S. deleyianum* [109], failed as the crystals began to disintegrate [156].

On the basis of crystallographic observations of the Fe(III)-NO_2_^−^ adduct and potential reaction intermediates, and of density functional calculations, a first working hypothesis for the reaction mechanism of NrfA, which hosts the novel Lys coordinated heme group (Fe-Lys), was developed by Oliver Einsle and Frank Neese (Fig. 8) [193]. Reduction of NO_2_^−^ started with the heterolytic cleavage of the N–O bond, facilitated by a pronounced back-bonding interaction of NO_2_^−^ coordinated through nitrogen to the reduced Fe(II) but not the oxidized Fe(III) of the active heme site. This step led to the formation of an {FeNO}^6^ species and a water molecule and was further facilitated by a hydrogen bonding network that induced an electronic asymmetry in the NO_2_^−^ molecule that weakened one N–O bond and strengthened the other. Subsequently, two rapid one-electron reductions led to an {FeNO}^8^ form and, by protonation, to an Fe(II)-HNO adduct. Hereafter, NH_2_OH, formed by a consecutive two-electron two-proton step, was dehydrated in the final two-electron reduction step to give NH_3_ and H_2_O. A single electron reduction of the active site closes the catalytic cycle [193]. In a set of consecutive theoretical studies, the NO_2_^−^ reduction mechanism was analysed in greater detail by Dimytro Bykov and Frank Neese (Max-Planck Institut für Chemische Energiekonversion, Mülheim) [194–197]. The mechanism comprises five functional stages. In phase 1, the substrate binds via its N atom to the active site heme 1 followed by N−O bond cleavage, with His 277 acting as the proton donor. In this step, the N−O bond of NO_2_^−^ is cleaved heterolytically through double protonation of the substrate. The second phase consists of two proton coupled electron-transfer events, leading to the HNO intermediate. Phase 3 involves the formation of NH_2_OH, where Arg 114 provides the necessary proton for the reaction. The second N−O bond is cleaved in phase 4 of the mechanism, again triggered by proton transfer from His 277. The Tyr 218 side chain governs the fifth and last phase of the mechanism, it consists of radical transfer and ammonia formation. In other words, this mechanism implies that all conserved active-site side chains work in a concerted way to achieve this complex chemical transformation from nitrite to ammonia. Interestingly, evidence for the active role of residue Tyr 218 was provided by earlier studies on the sulfite reductase activity of NrfAws and the active site variant Tyr218Phe. This NrfA variant exhibited an almost complete loss of nitrite reductase activity, while sulfite reduction remained unaffected [60]. According to the theoretical studies by Bykov and Neese, an intramolecular reaction with Tyr 218 in the final step of the nitrite reduction process led directly to the final product, NH_3_. Dissociation of the final product proceeds concomitantly with a change in heme Fe spin state [171, 197, 198].

Last but not least, recent reports surrounding the bioelectrochemical communication of enzymes, such as nitrogenase, nitrate reductase, or cytochrome *c* nitrite reductase, at electrode surfaces have demonstrated the ability to probe enzymatic mechanisms, to produce NH_3_ from a range of sources (e.g., NO_3_^−^, NO_2_^−^, N_3_^−^), and to detect biologically important N,O compounds, (e.g., NO_2_^−^, NO). Additionally, coupling of artificial cascade reactions have been utilized if a single catalyst is incapable of the complete substrate reduction to produce NH_3_ [199]. Electrochemical combined with spectroscopic techniques (UV/Vis, EPR, MCD) and site-directed mutagenesis, have been successfully applied to investigate multiheme enzymes by Julea Butt (Norwich), Sean Elliott (Boston), Kyle Lancaster (Ithaca), Andrew Pacheco (Milwaukee), and of course by Isabel, José Moura and their associates (Lisbon). The elegant studies by these leading researchers brought significant advances to our knowledge about important multi-electron, multi-proton transfer processes in biological systems [153, 166, 167, 173, 181, 200–212].

## Outlook and conclusions

Despite several decades of intensive research since the report by Fujita on soluble cytochromes in *Enterobacteriaceae* and the characterization of cytochrome *c*_552_ as a hexaheme nitrite reductase [63–65], there remain many unresolved issues concerning Nature’s nitrite to ammonia reductases. Clearly, cytochrome *c* nitrite reductase has become a mature field. The basic catalytic mechanism is understood at the atomic level in the context of electronic changes leading to NO_2_^−^ activation and its reduction to NH_3_/NH_4_^+^. Their catalytic versatility makes multiheme proteins and enzymes particularly valuable for numerous applications. Bioelectrochemical technology combined with various biophysical methods provide a new and powerful tool for a broad spectrum of biochemical and biophysical applications. Important structural and mechanistic information on multiheme proteins including extracellular electron transfer can be obtained by this technology, ranging from quaternary information, protein–protein and protein–surface recognition to highly resolved molecular pictures. In summary, *c*-type cytochromes will continue to be an important field of research. Determining and understanding their reaction mechanisms have greatly advanced a variety of fields and our understanding of activation of inorganic nitrogen and sulfur compounds as well as multi-electron and multi-proton transfer. Molecular dynamics simulations and quantum–mechanical/molecular-mechanical calculations will complement experiments and elucidate the choreography by which the cytochrome protein regulates the catalytic cycle [213–220].

Perhaps the exciting feature of the bacterial enzyme cytochrome *c* nitrite reductase (NrfA), first proposed by Jeff Cole and co-workers in 1998 on the basis of sequence studies for the nitrite reductase of *E. coli*, was the novel heme binding motif Cys-X-X-Cys-Lys (CXXCK) [161]. One year later, Oliver Einsle and associates presented the first high resolution NrfA structure of sulfur-reducing bacterium *S. deleyianum*, a homodimer with five covalently bound heme centers in each monomer, four of them carrying the conventional bis-His coordination (Cys-X-X-Cys-His motif), and the active site heme carrying the novel Cys-X-X-Cys-Lys motif, with a catalytically important Ca^2+^ cation above the distal side of the heme plane [162]. Today, we know from extensive investigations of the diversity and phylogeny of the NrfA enzyme including analysis of full-length NrfA protein sequences that there exist several clades carrying the conventional Cys-X-X-Cys-His motif in the first heme-binding domain [174–176], yet, detailed structural information on these enzymes by X-ray crystallography still has to come. The presence of the distal Lys residue was thought to be of crucial importance in promoting the binding of anions, and the design of the catalytic cavity around heme 1 was proposed to provide a reduction potential low enough for the reduction of nitrite to ammonium [183]. There exist naturally occurring heme proteins with Lys axially coordinated to Fe, such as the alkaline form of cytochrome *c* [221, 222], or the truncated hemoglobin THB1 of *Chlamydomonas reinhardtii* [223]. Furthermore, a Met100Lys variant of cytochrome *c*_550_ from *Paracoccus versutus* was produced, leading to a shift of the midpoint potential by − 329 mV compared to wild type [224, 225].

The His93Gly myoglobin cavity mutant was introduced by John Dawson and co-workers (University of South Carolina) as a valuable model system to investigate for endogenous Lys and terminal amine ligation in heme proteins. Replacement of proximal ligand His93 with the much smaller non-coordinating Gly residue left a cavity on the proximal side of the heme into which a wide variety of exogenous ligands could be delivered. The scaffold provided a remarkably versatile template for the preparation of model heme complexes and was used to mimic the heme iron coordination structure of native heme proteins, such as NrfA (Fe-N_Lys_), the CooA transcription factor (Fe-N_Pro_), or cytochrome *f* (Fe-N_Tyr_) [226, 227]. Along these lines, Nicholas Watmough and associates (University of East Anglia) created the His93Lys variant of sperm whale myoglobin and studied its binding and reactivity with nitrite [228]. Substitution of the proximal His ligand with Lys led to an eightfold increase in the rate of NO_2_^−^ → NO reduction relative to wild-type myoglobin. The binding of NO_2_^−^ via the oxygen atom (*O*-nitrito mode) to the Fe(III) heme was retained in His93Lys myoglobin. Nitrite can coordinate to heme iron through either nitrogen (*N*-nitrito mode), e.g., in cytochrome *c* nitrite reductase or in assimilatory and dissimilatory sulfite reductase, or through oxygen as observed for myoglobin and hemoglobin. This is known as linkage isomerism [229, 230]. Based on site-directed mutagenesis studies, it was argued that the His residue on the distal heme side modulates this unique *O*-nitrito binding mode. This suggestion received strong support by the computation of spin Hamiltonian EPR parameters of different linkage isomers of NO_2_^−^ bound myoglobin using wave function based “ab initio” and density functional theories [231].

Finally, the work by Tamara Tikhonova and colleagues (Russian Academy of Sciences) on related octaheme nitrite reductases should be mentioned here [232–235]. The first representative of this family of octaheme cytochrome *c* nitrite reductases was isolated from the haloalkaliphilic bacterium *Thioalkalivibrio nitratireducens*. The enzyme converted nitrite and hydroxylamine to ammonia without release of intermediates with high activity, as well as sulfite to sulfide. In solution, it exists as a stable hexamer, each subunit contains eight *c*-type hemes, seven of them are coordinated by the conventional CXXCH motif, while one, like in pentaheme NrfA, is bonded by the unique CXXCK motif.

In summary, the bioinorganic chemistry of multiheme *c*-type cytochromes is a vibrant field and will remain in the focus of active research in chemistry, biochemistry, microbiology and geochemistry, as impressively documented by discoveries of new enzymes with astounding chemical activities and novel active sites. Notably, the field of geomicrobiology has experienced an extraordinary growth in recent years, and microbes have been studied in all kinds of environments on Earth [113, 236]. In the context of *c*-type cytochromes, *Geobacter* bacteria are of high interest in the bioremediation and bioenergy fields, due to their ability to produce high current densities in microbial fuel cells, consequently, they are interesting targets for bioenergy applications. Closely related to these physiological features is the ability of *Geobacter* cells to produce conductive protein nanowires, a property that is currently being explored in the bionanotechnology field [122, 237].

## Abbreviations

DSIR: Dissimilatory sulfite reductase
EPR: Electron paramagnetic resonance
ET: Electron transfer
DNRA: Dissimilatory nitrate reduction to ammonium
HAO: Octaheme hydroxylamine oxidoreductase
HDH: Hydrazine dehydrogenase
MCD: Magnetic circular dichroism
NMR: Nuclear magnetic resonance
NrfA: Pentaheme cytochrome *c* nitrite reductase
NrfAec: Nitrite reductase of *Eschericia coli*
NrfAgl: Nitrite reductase of *Geobacter lovleyi*
NrfAsd: Nitrite reductase of *Sulfurospirillum deleyianum*
NrfAws: Nitrite reductase of *Wolinella succinogenes*
PDB: Protein data bank; https://www.rcsb.org
UV/Vis: Ultraviolet/visible
